# HFE Gene Mutations, Iron Overload and Cryptogenic Liver Cirrhosis

**DOI:** 10.5812/hepatmon.824

**Published:** 2012-03-28

**Authors:** Christoph Eisenbach

**Affiliations:** 1 Department of Gastroenterology, University Hospital of Heidelberg, Heidelberg, Germany

**Keywords:** Genes, Iron, Liver

Dear Editor,

The diagnosis of cryptogenic cirrhosis is an exclusion diagnosis. It has become far less frequent over the last decades, but it still effects a significant number of patients. Many previously unknown chronic liver disease entities were described in the second half of the last century, including chronic viral hepatitis B, C and D, along with refined criteria for diagnosing autoimmune hepatitis and insights into the pathogenesis and disease progression of fatty liver disease [1]. Although hemochromatosis, and its pathophysiology were not understood until cloning of the HFE-gene [2], it has been known as a chronic liver disease for a long time, although it may be under-recognized due to the lack of specific symptoms and thus contribute to cases of cryptogenic cirrhosis.

Jowkar et al. [3] described the frequency of the most common disease-causing mutations of the HFE-gene (C282Y and H63D) in Iranian patients with cryptogenic cirrhosis and healthy controls. They did not observe any C282Y mutations and only heterozygous H63D mutations were found in about one quarter of both healthy and cirrhotic patients. As the C282Y mutation is most likely to be of northern European origin, the low frequency found in the present report is not surprising. The H63D mutation however, was present heterozygous in almost one quarter of the patients without any of these patients carrying homozygous mutations, this is surprising and much higher than would be expected [4][5]. As there were no differences between healthy controls and cirrhotic patients, these numbers most likely reflect the allele frequency in the general Iranian population and were described for the first time in the current report. The authors present results for patients with and without iron overload. Unfortunately, the numbers of patients diagnosed with iron overload are not reported. Therefore, the relative proportion of patients carrying the H63D mutation cannot be calculated and the impact of iron overload on cryptogenic cirrhosis cannot be deduced. Furthermore, iron overload in the paper by Jowkar et al. has been defined by transferrin saturation. It would be valuable to know serum-ferritin levels in order to get further insights into the state of iron metabolism. Classic HFE related hemochromatosis, as demonstrated by the authors, is extremely unlikely in this population. If iron overload were indeed present it would be worthwhile testing for other hemochromatosis associated mutations, namely transferrin receptor 2 (tfr2) and ferroportin (fpn) [6].

In conclusion, Jowkar et al. confirmed the low frequency of hemochromatosis causing HFE mutations in the Iranian population for the first time. Further studies are still needed to evaluate the impact of hereditary iron metabolism disorders on cryptogenic cirrhosis in Iran.
